# Supplementary material for: Transcriptional and spatial profiling of the kidney allograft unravels a central role for FcyRIII+ innate immune cells in rejection
Source: Nat Commun. 2023 Jul 19;14:4359. doi: 10.1038/s41467-023-39859-7 (PMC10356785; doi:10.1038/s41467-023-39859-7)
Supplement: Supplementary file 1 — Supplementary Information [file 41467_2023_39859_MOESM1_ESM.pdf]

Transcriptional and spatial profiling of the kidney allograft unravels a central role for FcγRIII+ innate immune cells in rejection

Baptiste Lamarthée[1,2], Jasper Callemeyn[1,3], Yannick Van Herck[4], Asier Antoranz[5], Dany Anglicheau[6,7], Patrick Baoda[8], Jan Ulrich Becker[9], Tim Debyser[1], Frederik De Smet[5] , Katrien De Vusser[1,3], Maëva Eloudzeri[7], Amelie Franken[10,11] , Wilfried Gwinner[12], Priyanka Koshy[13], Dirk Kuypers[1,3], Diether Lambrechts[10,11], Pierre Marquet[14], Virginie Mathias[15,16], Marion Rabant[7,17], Minnie M. Sarwal[8], Aleksandar Senev[1,18], Tara K. Sigdel[8], Ben Sprangers[1,3] , Olivier Thaunat[16,19], Claire Tinel[1,2,20], Thomas Van Brussel[10,11], Amaryllis Van Craenenbroeck[1,3], Elisabet Van Loon[1,3], Thibaut Vaulet[1], Francesca Bosisio[5], Maarten Naesens[1,3]

Affiliations

[1]Department of Microbiology, Immunology and Transplantation, Nephrology and Kidney Transplantation Research Group, KU Leuven, Leuven, Belgium

[2]Université de Franche-Comté, UBFC, Inserm UMR1098 Right, EFS BFC, Dijon, France

[3]Department of Nephrology and Kidney Transplantation, University Hospitals Leuven, Leuven, Belgium

[4]Department of Oncology, Laboratory for Experimental Oncology, KU Leuven, Leuven, Belgium

[5]Department of Imaging and Pathology, Translational Cell and Tissue Research, KU Leuven, Leuven, Belgium

[6]Department of Nephrology and Kidney Transplantation, Necker-Enfants Malades Hospital, Assistance Publique-Hôpitaux de Paris, Paris, France

[7]Université Paris Cité, Inserm U1151, Necker Enfants-Malades Institute, Paris, France

[8] Division of Multi-Organ Transplantation, Department of Surgery, UCSF, 513 Parnassus, San Francisco, California, United States

[9]Institute of Pathology, University Hospital Cologne, Cologne, Germany

[10]VIB Center for Cancer Biology, Leuven, Belgium

[11]Department of Human Genetics, Laboratory of Translational Genetics, KU Leuven, Leuven, Belgium

[12]Department of Nephrology, Hannover Medical School, Hannover, Germany

[13]Department of Imaging and Pathology, KU Leuven, Leuven, Belgium;

[14]Department of Pharmacology and Transplantation, University of Limoges, Inserm U1248, Limoges University Hospital, Limoges, France

[15]EFS, HLA Laboratory, Décines, France

[16] Université Claude Bernard Lyon I, Inserm U1111, CNRS UMR5308, CIRI, Ecole Normale Supérieure de Lyon, Lyon, France.

[17]Department of Pathology, Necker-Enfants Malades Hospital, Assistance Publique-Hôpitaux de Paris, Paris, France

[18]Histocompatibility and Immunogenetics Laboratory, Red Cross-Flanders, Mechelen, Belgium

[19] Hospices Civils de Lyon, Edouard Herriot Hospital, Department of Transplantation, Nephrology and Clinical Immunology, Lyon, France.

[20] Department of Nephrology and Kidney Transplantation, Dijon Hospital, Dijon, France

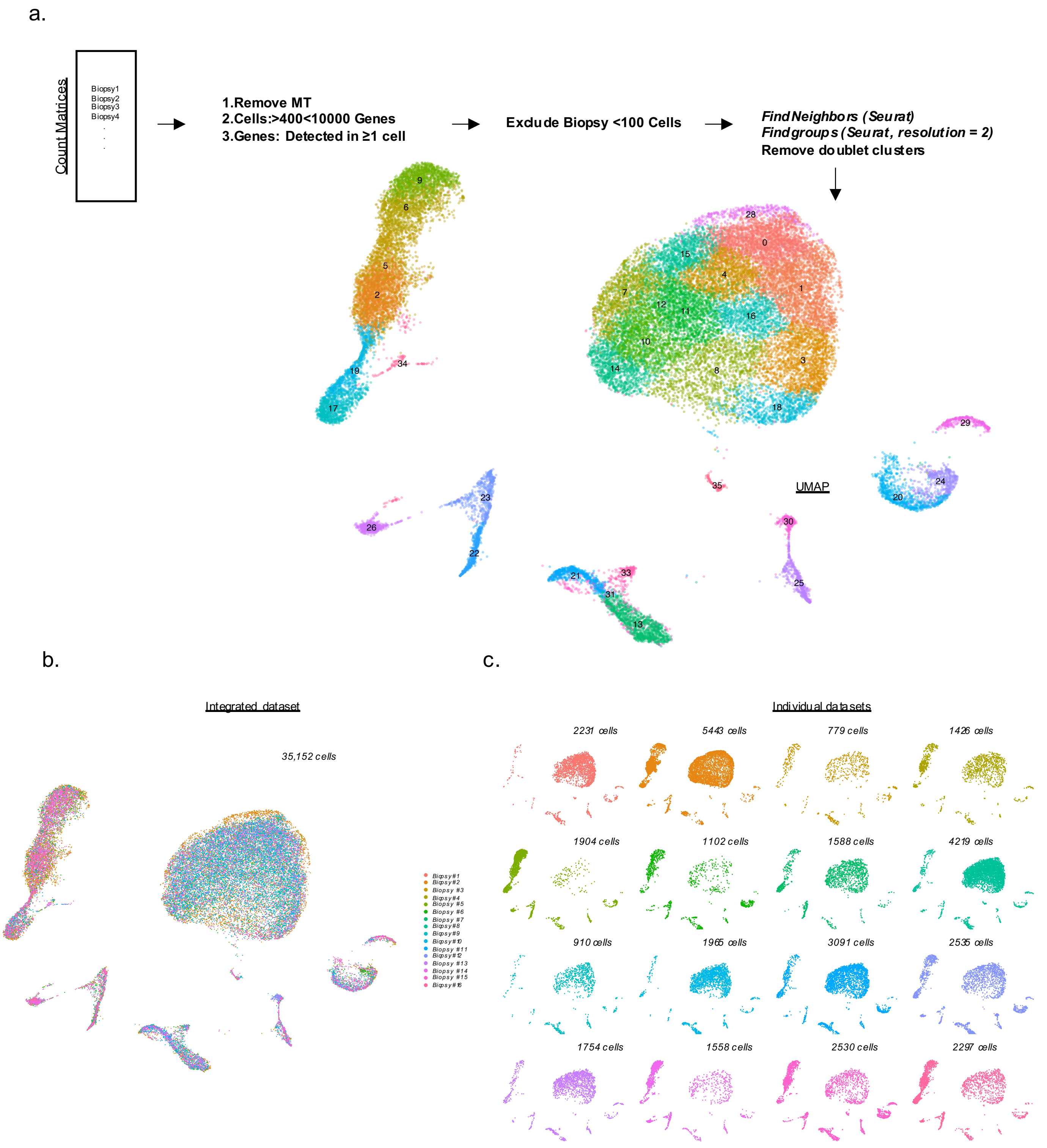

Figure S1 Quality control of the scRNA-seq dataset. a) Quality control and parameters of the generation of the single cell dataset b) UMAP visualization of the whole dataset grouped by original biopsy identity. c) UMAP visualization of each individual biopsy in the dataset demonstrating equal coverage of all cell clusters. MT, a set of mitochondrial genes.

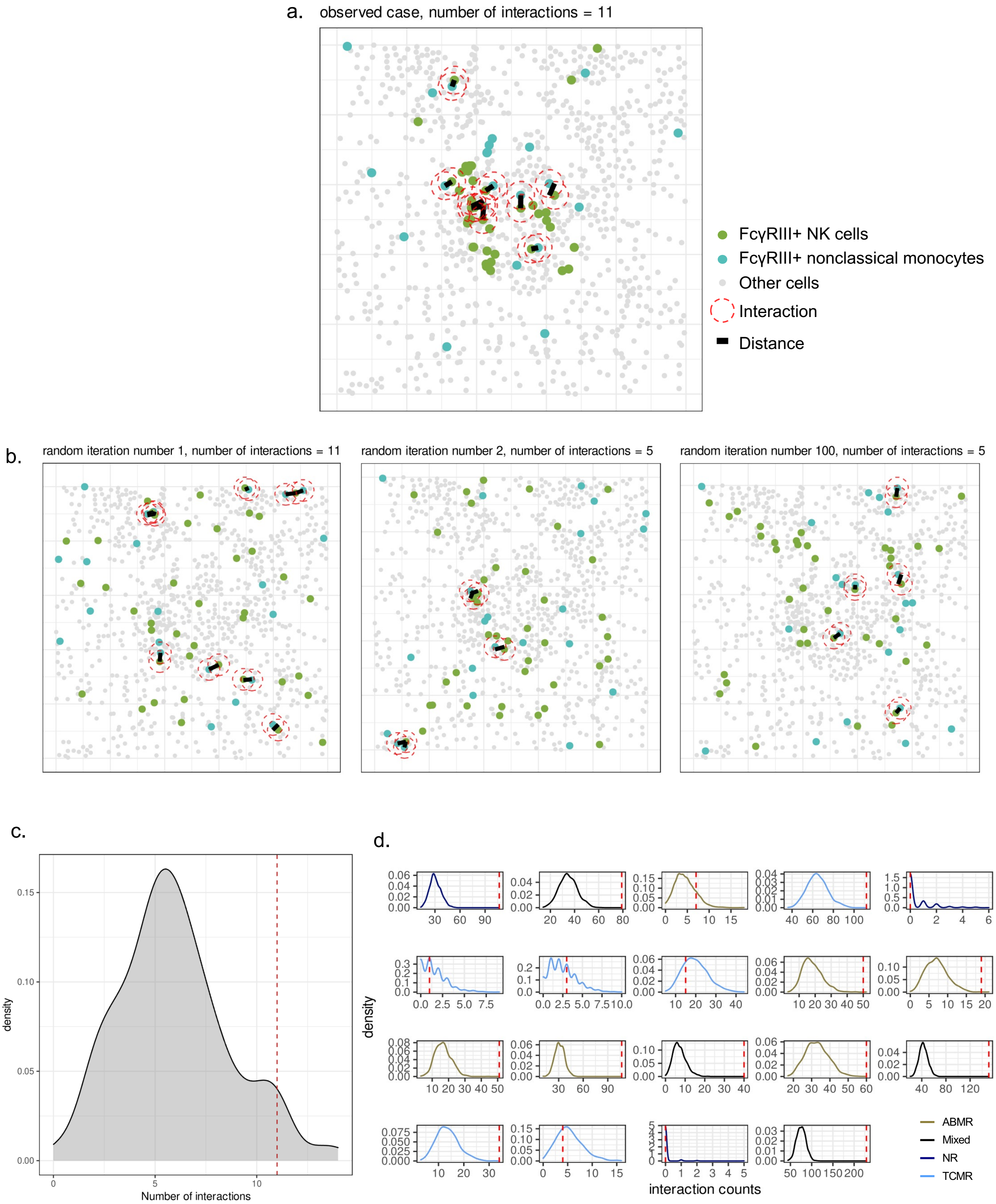

Figure S2 Neighborhood analysis: intermediate results. a) Scatter plot showing the location of FcγRIII+ NK cells (green), FcγRIII+ nonclassical monocytes (turquoise), and other cell types (gray) in a preselected tissue area for the observed data. FcγRIII+ NK cells in the neighborhood of FcγRIII+ nonclassical monocytes are represented by red dashed circles and connecting black lines. b) The same tissue area is represented after randomly permutating the label of each cell type. The reader should note that both the location of each cell as well as the number of cells of each type have been preserved. Only their labels have been randomly permutated. This process is repeated 1000 times. Here iteration numbers 1 (left), 2 (center), and 100 (right) are represented. c) Density plot showing the distribution of counts found in all 1000 random iterations. The vertical red dashed line instead represents the number of counts found in the observed data. The relative number of random cases with more counts than the observed cases can be interpreted as an empirical p-value representing the significance of a cell-cell interaction. d) The same exercise was repeated for each individual sample taking into account the whole tissue. These density plots represent an intermediate step towards the plot shown in Figure 9C.

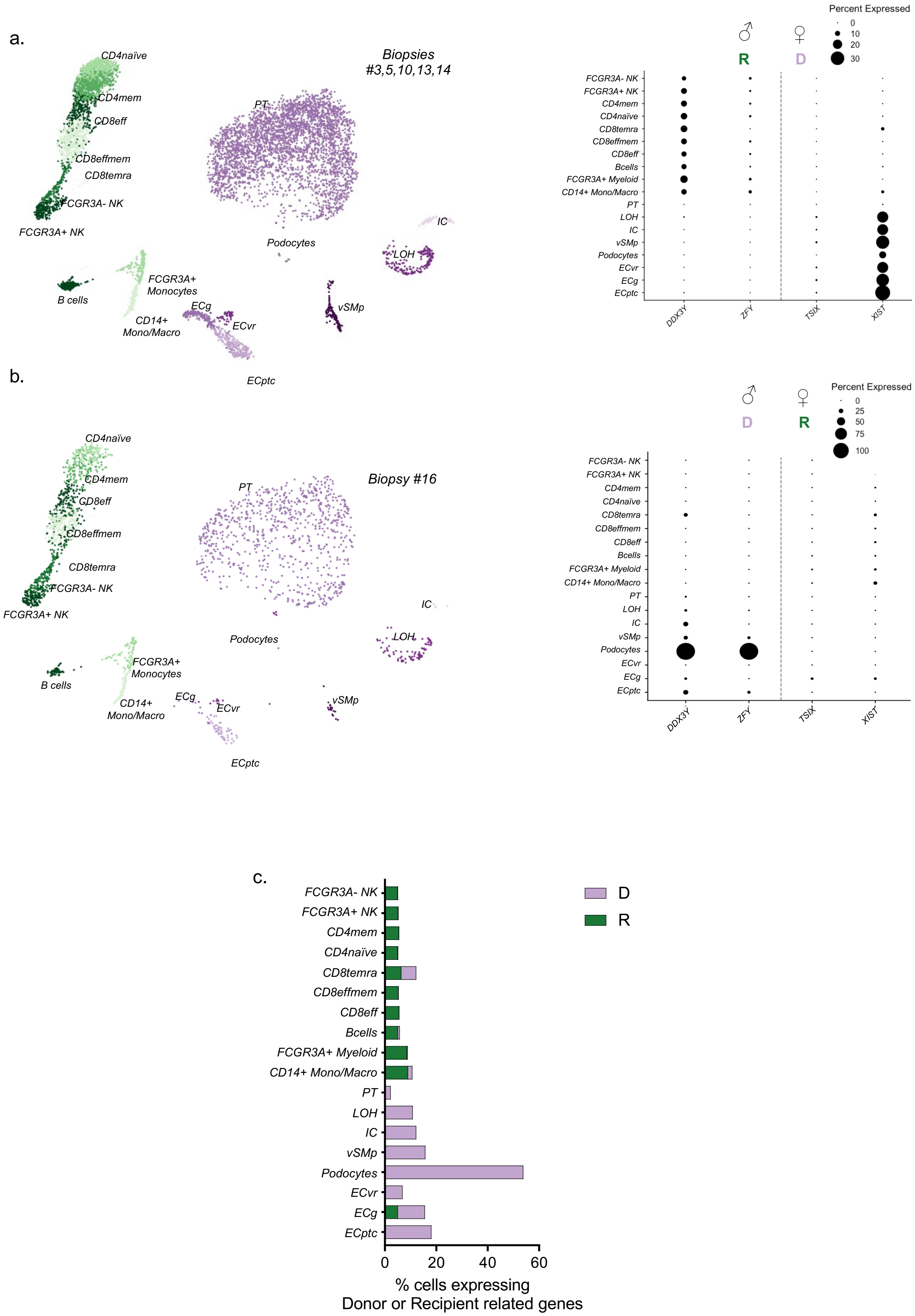

Figure S3 Origin of the cells (a-b left panels) UMAP visualization of the cells corresponding to the five biopsies derived from a female donor and a male recipient or to the single biopsy derived from a male donor and a female recipient (a-b right panels) Dot plot showing the expression of sex-related genes.

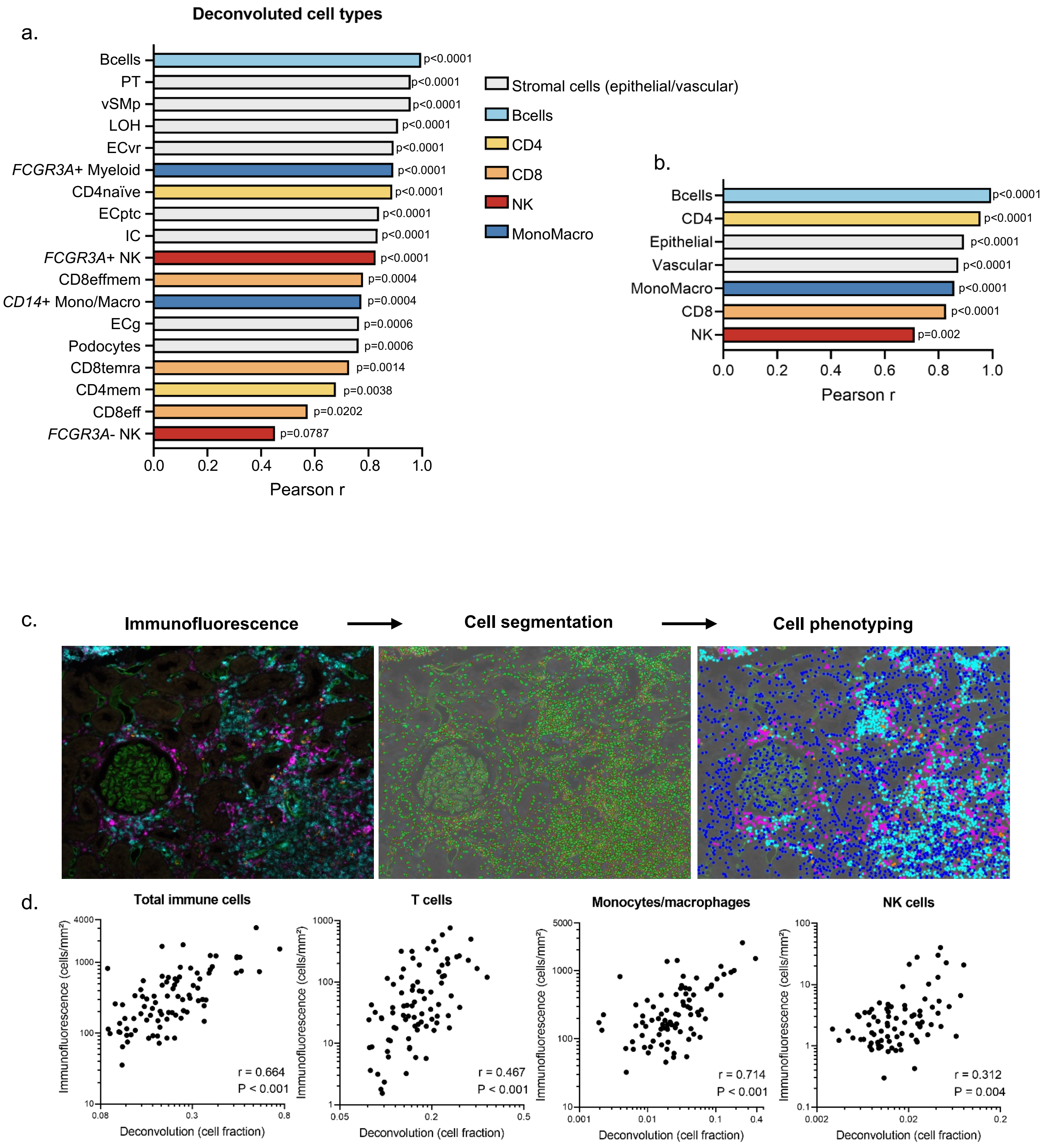

Figure S4 Validation of deconvolution results a) Correlation of deconvoluted cell populations based on pseudobulk analysis of single cell RNA seq samples (n=16) and actual cell counts in the sample. b) Correlation as described in a) for broader defined cell types. Two tailed Pearson correlation test were performed and p values were depited c) Workflow of OPAL immunofluorescence imaging and subsequent computerized segmentation, phenotyping and quantification. d) Pearson's correlation between the deconvoluted cell populations based on microarray data and the quantified populations using OPAL imaging.

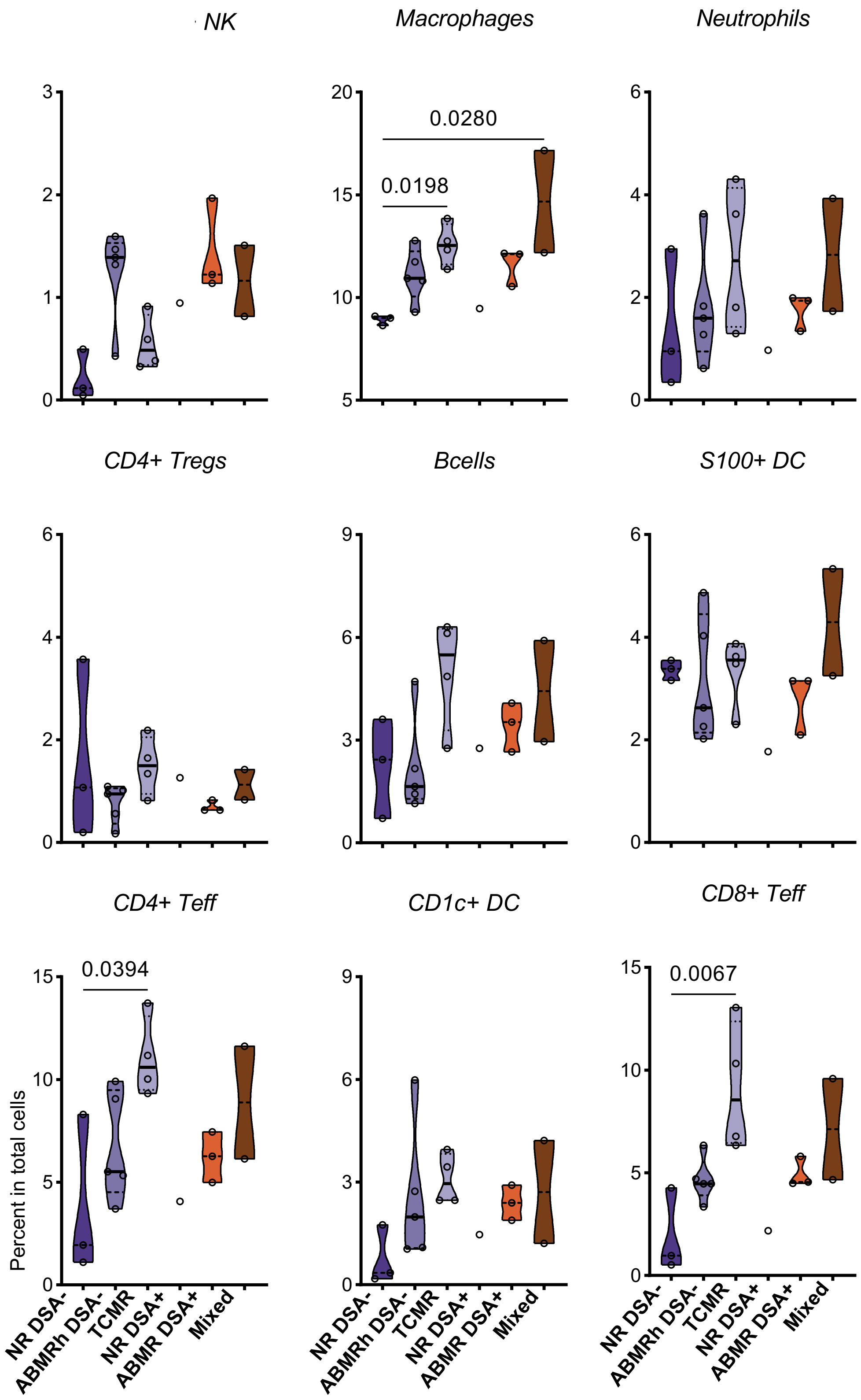

Figure S5 Proportion of cells according to Banff Classification. Violin plots depicting the proportion of indicated cells measured by MILAN method regarding clinical outcome. The difference between groups was assessed by a Kruskal-Wallis test and multiple comparisons using the Dunn's test.

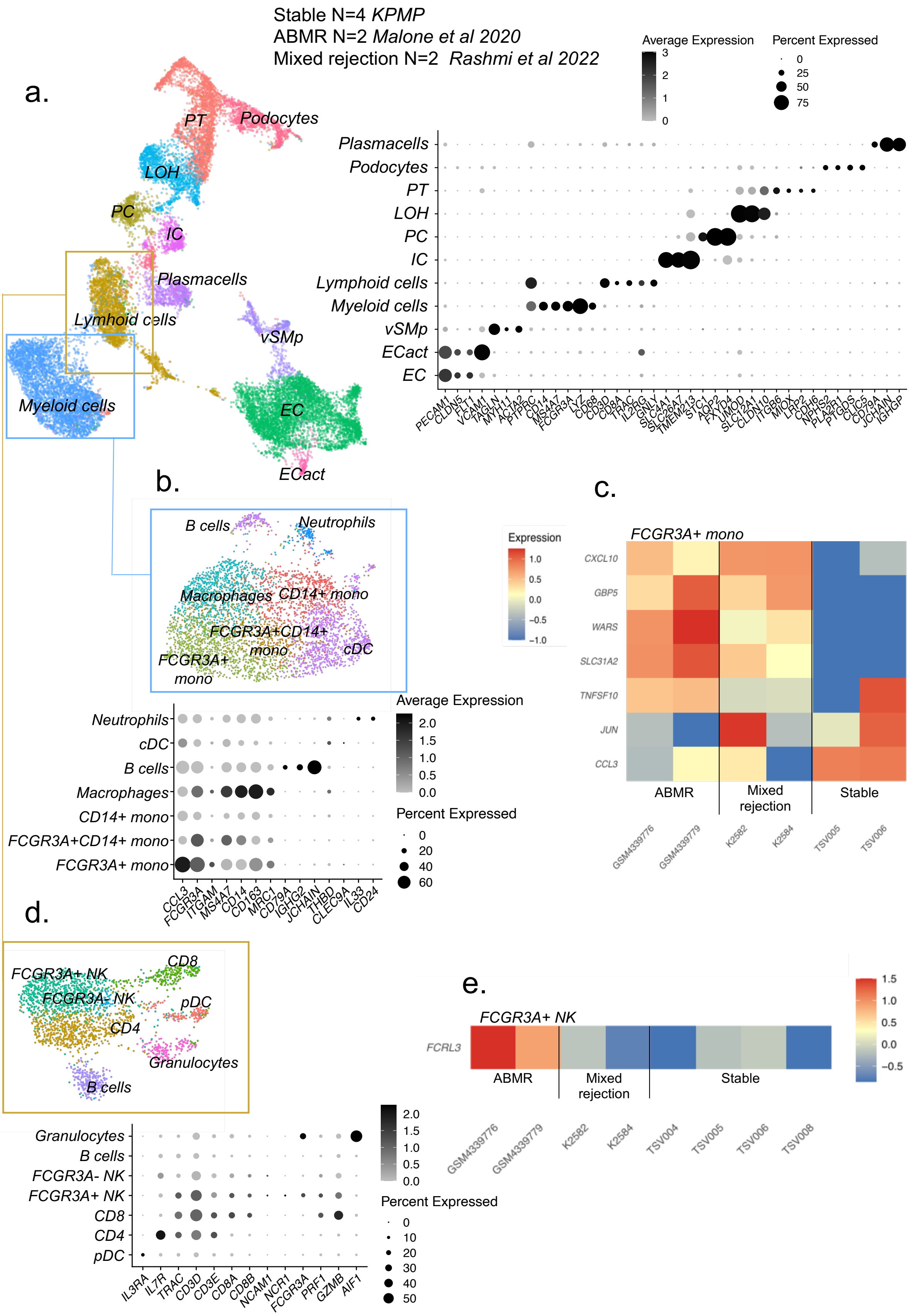

Figure S6 Validation using an external single-cell RNAseq dataset. a) Public data derived from kidney biopsies were analyzed. Datasets derived from two mixed rejection samples, 2 ABMR samples and 4 stables samples (KPMP repository) were reintegrated. UMAP and Dot plot showing identifying markers of each cell type. b) Myeloid cells were subclustered and reintegrated. UMAP and Dot plot showing identifying markers of each myeloid cell type. c) Heatmap showing the expression of indicated genes in *FCGR3A+ CD14-* monocytes is depicted.d) Lymphoid cells were subclustered and reintegrated. UMAP and Dot plot showing identifying markers of each lymphoid cell type. e) Heatmap showing the expression of indicated gene in *FCGR3A+* NK is depicted.

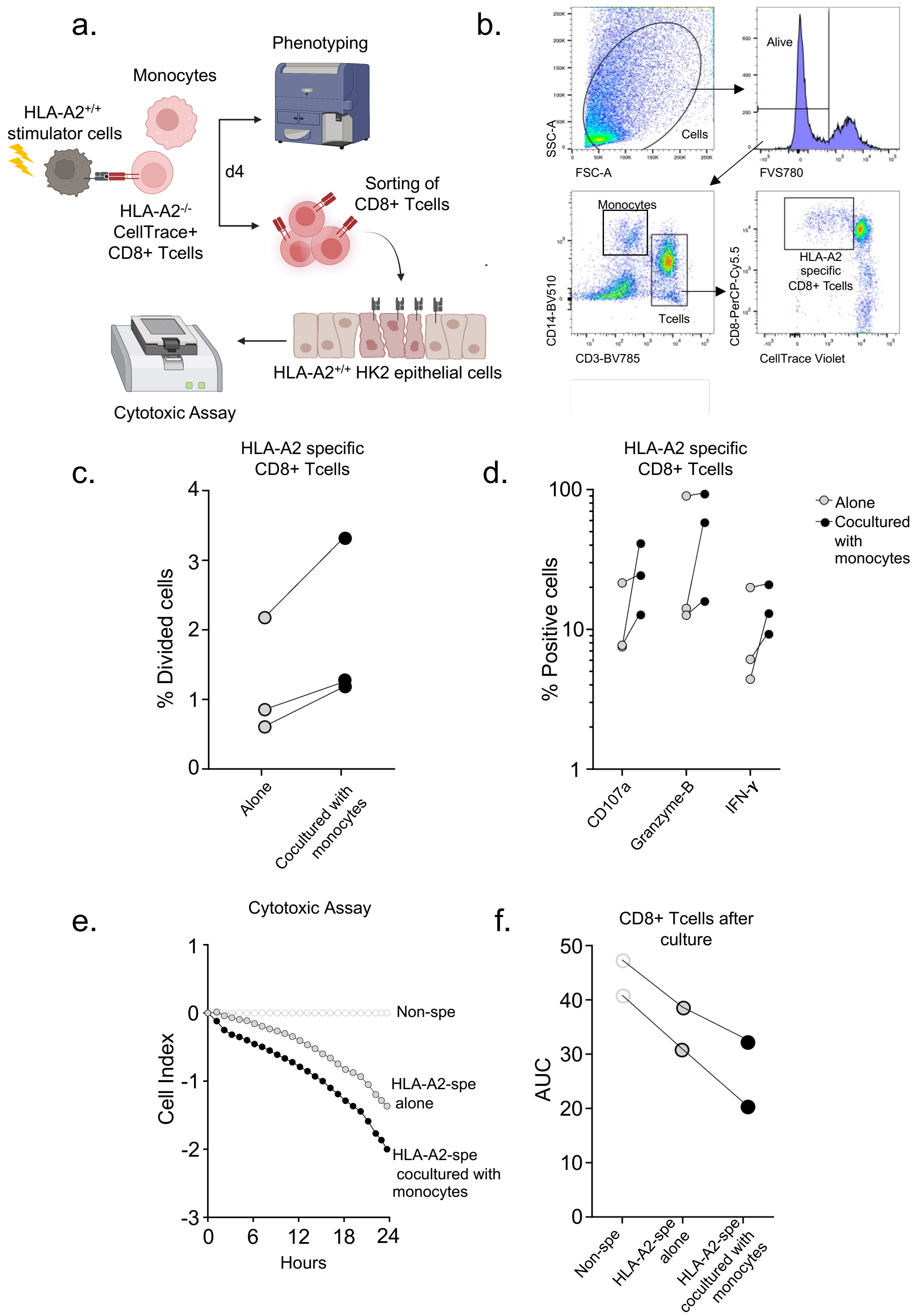

Figure S7 Monocytes-CD8+T cells collaboration a-f) HLA-A2$^{-/-}$ total monocytes and CD8+ Tcells were cocultured and activated with an irradiated HLA-A2+/+ human stimulator cell line. After 4 days of culture, cells were harvested and CD8+ T cells were either stained for cytometry analysis or purified for cytotoxic assay. a) Experimental scheme created using Biorender.com b) Gating strategy: the cells were selected based on FSC/SSC parameters. Exclusion of dead cells was performed by selection of FVD negative cells. Monocytes were gated based on CD14-BV510 positivity whereas T cells were selected using CD3-BV786 marker. Ultimately, CD8+ T cells were selected using CD8-PerCP-CY5.5 marker c) Percent of CellTrace negative divided HLA-A2 specific CD8+ Tcells is depicted from N=3 independent experiments. d) Percentage of HLA-A2 specific CD8+Tcells positive for indicated markers is depicted from N=3 independent experiments. e-f) After coculture with or without monocytes, CD8+ Tcells were purified and their cytotoxic capacity was monitored for 24h against HLA-A2+/+ HK2 target cells. N=2 independent experiments e) Cell index is depicted f) Area Under the Curve (AUC) is depicted.

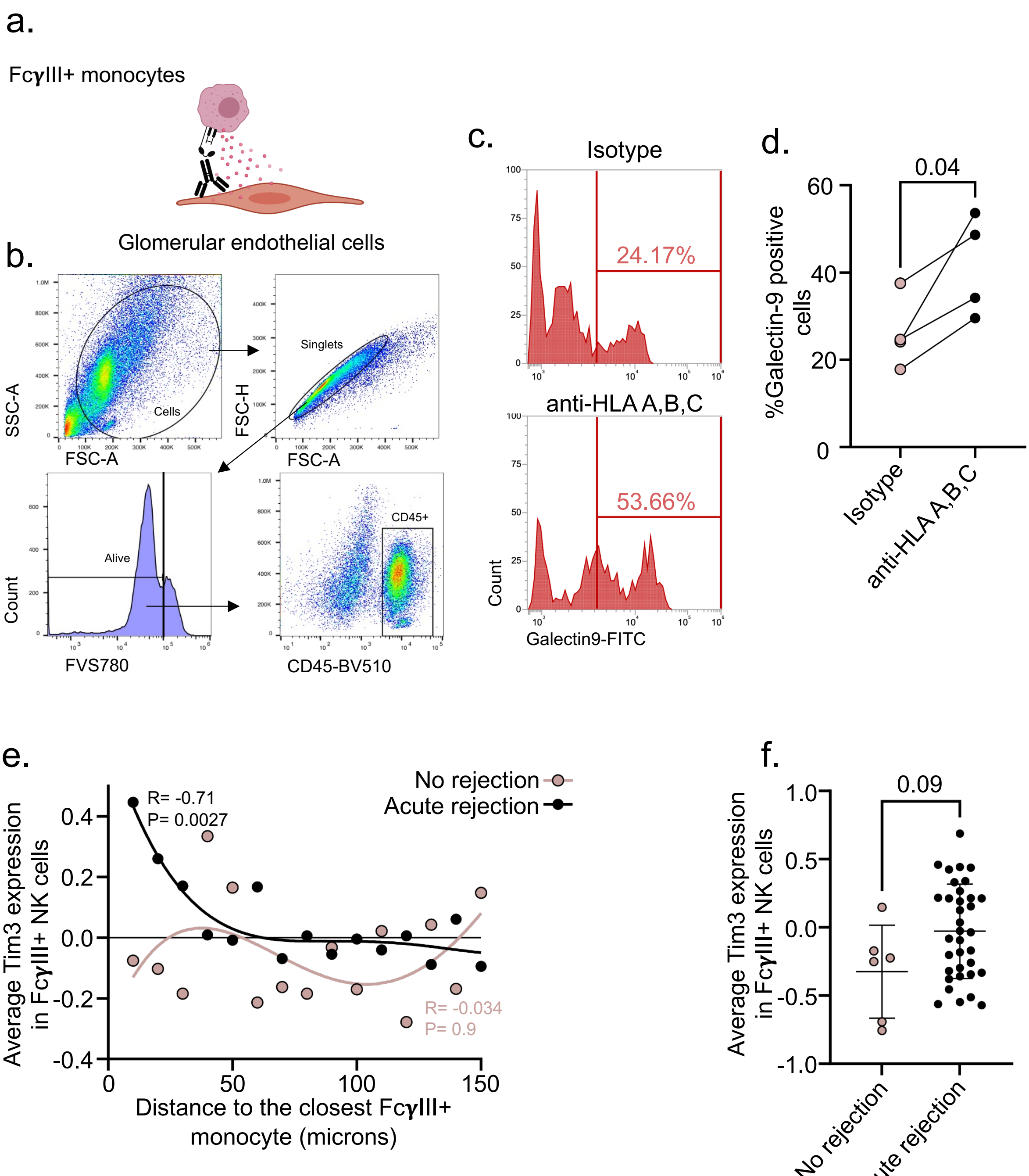

Figure S8 Exploration of LGALS9-HAVCR2 communication- a-d) FcγIII+ monocytes were isolated from a healthy volunteer for *in vitro* coculture with anti HLA A,B,C or isotype and glomerular endothelial cells (GENC) as previously described for 36h. a) After 36h of culture, galectin 9 secretion was measured by flow cytometry. Experimental scheme created using Biorender.com b) Gating strategy. the cells were selected based on FSC/SSC parameters. Exclusion of doublets was performed using FSC-H/FSC-A parameters. Dead cells exclusion was performed by selection of FVD negative cells. Immune cells (monocytes) were gated based on CD45-BV510 positivity. Ultimately, Galectin9+ monocytes were selected using Galectin9-FITC marker c-d) The proportion of galectin 9 positive cells in total living CD45+ cells was depicted in histograms c) and scatter plot d) and was significantly increased in anti HLA A,B,C condition as assessed by the two-tailed p value calculated with a Mann-Whitney test. N=4 independent experiments e-f) Tim3 expression in FcγIII+ NK cells was analyzed using MILAN technology. e) Tim3 expression in NK cells was assessed in biopsies showing no rejection as compared to acute rejection according to the distance to the closest FcγIII+ monocyte. The correlations were compared using a two-tailed Pearson test. f) The average expression of Tim3 FcγIII+ NK present in the regions of interest of biopsies was compared between groups. The mean ± standard deviation is depicted. The difference between groups was assessed by a two-tailed Mann-Whitney test.

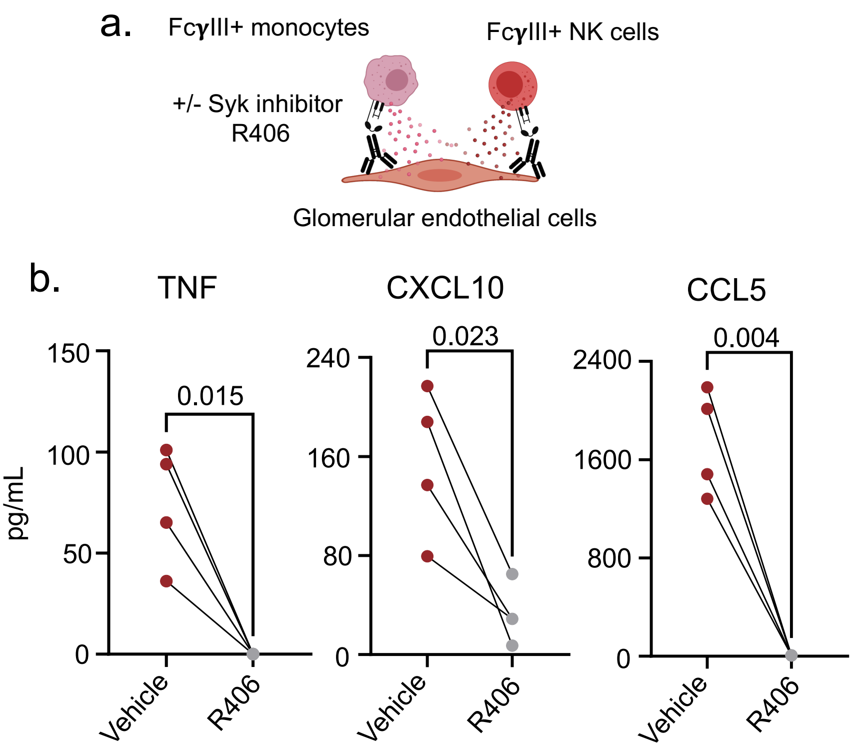

Figure S9 Syk inhibition strongly dampens proinflammatory cytokines secretion by cγlll+ monocytes and Fcγlll+ NK cells Fcγlll+ monocytes and Fcγlll+ NK cells were isolated from a healthy volunteer for *in vitro* coculture with anti HLA A,B,C or isotype and glomerular endothelial cells (GENC) for 36h a) Fcγlll+ monocytes and Fcγlll+ NK cells were cultured with GENC previously incubated with anti HLA A,B,C for 36h. When indicated, R406 (2μM) or vehicle (DMSO) was added to the coculture. Experimental scheme created using Biorender.com (b) The concentration of TNF, CXCL10 and CCL5 was measured by Cell Bead Array in the culture supernatant and depicted in scatter plots. The difference between groups was assessed by the two-tailed p value calculated with paired t tests. N=4 independent experiments

Supplementary tables

Table S1. Clinical and pathological features of the scRNAseq study population. DBD: donation after brain death, DCD: donation after circulatory death, LD: living donation, M: male, F: female, HLA-DSA: donor-specific anti-human leukocyte antigen antibodies, NR: no rejection, ABMR: antibody-mediated rejection, TCMR: T cell-mediated rejection, *Biopsies from the same patient, **Biopsies from the same patient

**Biopsy no.**	**Recipient age at tx**	**Indication/protocol**	**Days after tx**	**Donation type**	**Recipient and donor gender**	**Induction**	**Serum creatinine (mg/dl)**	**HLA-DSA**	**Histological lesions according to the Banff criteria**	**Histological biopsy phenotype according to Vaulet et al[1]**
#1	34	Indication	37	DBD	M/M	Basiliximab	1.76	A11	g0, ptc0, C4d0, v0, cg0, t0, i0, ct0, ci0	NR DSA+
#2*	52	Indication	61	DCD	F/F	None	4.46	DPB1*03:01 (104:01)	g2, ptc2, C4d2, v0, cg0, t0, i1, ct1, ci0	ABMR
#3	45	Indication	12	LD	M/F	Basiliximab	2.09	DQ1 (DQA1*01:04/DQB1*05)	g0, ptc0, C4d0, v0, cg0, t1, i1, ct0, ci0	NR DSA+
#4*	52	Protocol	91	DCD	F/F	None	1.92	DPB1*03:01 (104:01)	g1, ptc1, C4d1, v0, cg0, t0, i0, ct0, ci0	NR DSA+
#5	58	Indication	9	DBD	M/F	Basiliximab	1.86	B62, B35	g1, ptc2, C4d0, v1, cg0, t0, i0, ct0, ci0	NR DSA+
#6	11	Indication	7709	DBD	M/M	Thymoglobulin	8.08	No HLA-DSA	g0, ptc0, C4d0, v0, cg0, t0, i0, ct2, ci2	NR DSA-
#7	55	Indication	6	DBD	M/M	Basiliximab	6.93	DRB1*08:02	g2, ptc2, C4d1, v1, cg0, t1, i1, ct1, ci1	ABMR
#8	61	Indication	6	DCD	F/F	None	5.03	No HLA-DSA	g0, ptc2, C4d1, v0, cg0, t1, i1, ct0, ci0	TCMR
#9	19	Protocol	91	LD	M/M	Basiliximab	1.04	No HLA-DSA	g0, ptc0, C4d1, v0, cg0, t0, i0, ct0, ci0	NR DSA-
#10	57	Indication	44	DCD	M/F	Basiliximab	3.94	No HLA-DSA	g0, ptc0, C4d1, v0, cg0, t0, i0, ct1, ci1	NR DSA-
#11**	52	Indication	247	DCD	F/F	Basiliximab	1.87	DQ1 (DQB1*05:01 DQA1*01:01)	g0, ptc0, C4d0, v0, cg0, t0, i0, ct1, ci1	NR DSA+
#12	50	Indication	2104	DCD	M/M	Basiliximab	2.64	A32	g1, ptc0, C4d0, v0, cg0, t0, i0, ct1, ci1	NR DSA+
#13	63	Indication	118	DCD	M/F	Basiliximab	2.27	A23, DR7	g0, ptc0, C4d2, v0, cg0, t1, i1, ct1, ci1	NR DSA+

#14	49	Indication	6	DBD	M/F	Basiliximab	6.23	No HLA-DSA	g0, ptc0, C4d0, v0, cg0, t1, i1, ct1, ci1	NR DSA-
#15**	52	Indication	361	DCD	F/F	Basiliximab	3.10	DQ1 (DQB1*05:01 DQA1*01:01)	g0, ptc1, C4d0, v0, cg0, t1, i1, ct2, ci2	NR DSA+
#16	22	Indication	2002	DBD	F/M	Basiliximab	1.93	Cw6 DQ5	g2, ptc2, C4d3, v0, cg1, t0, i0, ct2, ci2	ABMR

Table S2. Clinical and pathological features of the Multiple Iterative Labeling by Antibody Neodeposition (MILAN) study population

**Biopsy no.**	**Recipient age at tx**	**Indication/protocol**	**Days** after **tx**	**Donation type**	**Recipient and donor gender**	**Induction**	**Serum creatinine (mg/dl)**	**HLA-DSA**	**Histological lesions according to the Banff criteria**	**Histological biopsy phenotype according to Vaulet et al**[1]
#1	67	Indication	16	DBD	M/F	Basiliximab	4.47	No HLA-DSA	g3, ptc1, C4d1, v0, cg0, t1, i0, ct0, ci0	ABMR DSA-
#2	37	Indication	5	DBD	M/M	Basiliximab	6.92	No HLA-DSA	g3, ptc2, C4d3, v0, cg0, t3, i2, ct0, ci0	ABMR DSA-
#3	58	Indication	70	LD	M/F	Basiliximab	2.87	DR7	g0, ptc0, C4d0, v0, cg0, t0, i0, ct1, ci0	NR DSA+
#4	37	Indication	19	DBD	M/M	Basiliximab	2.33	No HLA-DSA	g3, ptc1, C4d3, v0, cg0, t1, i0, ct0, ci0	ABMR DSA-
#5	29	Protocol	96	DBD	F/F	Basiliximab	0.92	B60<br>DQA1	g3, ptc0, C4d3, v0, cg0, t0, i0, ct1, ci0	ABMR
#6	57	Indication	59	DBD	M/M	None	3.65	No HLA-DSA	g0, ptc0, C4d0, v0, cg0, t0, i0, ct1, ci0	NR DSA-
#7	25	Indication	276	DBD	M/M	Basiliximab	1.45	DPB1*03:01<br>DPB1*04:01	g2, ptc3, C4d0, v0, cg1, t1, i2, ct1, ci0	ABMR
#8	71	Indication	6	DBD	M/F	None	3.95	No HLA-DSA	g0, ptc0, C4d0, v1, cg0, t2, i2, ct1, ci0	TCMR
#9	41	Indication	6	DBD	M/F	Basiliximab	10.23	A2	g2, ptc2, C4d1, v1, cg0, t2, i3, ct1, ci1	Mixed
#10	29	Protocol	1825	DBD	M/M	Basiliximab	1.46	No HLA-DSA	g0, ptc0, C4d0, v0, cg0, t2, i2, ct1, ci1	TCMR
#11	45	Protocol	371	LD	F/F	Basiliximab	1.70	No HLA-DSA	g0, ptc0, C4d0, v0, cg0, t2, i2, ct0, ci0	TCMR
#12	54	Protocol	363	LD	F/M	Basiliximab	2.15	No HLA-DSA	g2, ptc1, C4d0, v1, cg0, t1, i1, ct1, ci0	ABMR DSA-
#13	57	Protocol	374	DBD	F/F	Basiliximab	1.13	No HLA-DSA	g2, ptc2, C4d0, v0, cg0, t0, i0, ct2, ci2	ABMR DSA-
#14	47	Indication	9	DBD	F/F	Basiliximab	3.08	No HLA-DSA	g3, ptc0, C4d1, v1, cg0, t2, i3, ct0, ci0	ABMR DSA-
#15	56	Indication	172	DCD	F/M	Basiliximab	2.41	DPA1*02	g3, ptc0, C4d0, v1, cg0, t2, i3, ct0, ci0	Mixed
#16	69	Indication	5	DBD	M/F	None	1.80	No HLA-DSA	g0, ptc0, C4d0, v0, cg0, t2, i3, ct0, ci0	TCMR

#17	52	Indication	61	DCD	F/F	None	4.46	DPB1*03:01 (104:01)	g2, ptc2, C4d2, v0, cg0, t0, i1, ct1, ci0	ABMR
#18	19	Protocol	91	LD	M/M	Basiliximab	1.04	No HLA-DSA	g0, ptc0, C4d1, v0, cg0, t0, i0, ct0, ci0	NR DSA-

Table S3. Antibodies used for MILAN. AF; Alexa Fluor

Marker	Analysis	Reference	Supplier	Host species	Clone	Dilution
AQP1	Phenotypic identification	AB2219	Sigma Aldrich	Rabbit	Polyclonal	1:20000
CD1c	Phenotypic identification/Monocyte subclustering	UM500042	OriGene	Mouse IgG1	UMAB46	1:333
CD3	Phenotypic identification	MA1-90582	ThermoFisher	Rabbit	SP7	1:200
CD4	Phenotypic identification	ab133616	Abcam	Rabbit	EPR6855	1:166
CD8	Phenotypic identification	sc-53212	Santa Cruz Biotechnology	Mouse IgG1	C8/144B	1:500
CD11b	Monocyte subclustering	ab133357	Abcam	Rabbit	EPR1344	1:5000
CD11c	Phenotypic identification/Monocyte subclustering	sc-46676	Santa Cruz Biotechnology	Mouse IgG1	B-6	1:75
CD14	Phenotypic identification/Monocyte subclustering	#75181	Cell Signaling Technology	Rabbit	D7A2T	1:200
FcγIII	Phenotypic identification/Monocyte subclustering	NCL-L-CD16	Leica Biosystems	Mouse IgG2a	2H7	1:333
CD20	Phenotypic identification	M075501-2	Agilent	Mouse IgG2a	L26	1:630
CD31	Exploratory Analysis	LS-C173974	LSBio	Mouse IgG2a	OTI2C6	1:1000
CD56	Phenotypic identification	sc-7326	Santa Cruz Biotechnology	Mouse IgG1	123C3.D5	1:200
CD57	Phenotypic identification	MAB8560	R&D Systems	Mouse IgG2a	1002707	1:250
CD68	Phenotypic identification/Monocyte subclustering	MA5-12407	ThermoFisher	Mouse IgG3	PGM1	1:200
CD69	Exploratory Analysis	HPA050525	Sigma Aldrich	Rabbit	Polyclonal	1:50
CD79a	Exploratory Analysis	sc-53209	Santa Cruz Biotechnology	Mouse IgG1	JCB117	1:2000
CD123	Monocyte subclustering	NCL-L-CD123	Leica Biosystems	Mouse IgG2b	BR4MS	1:10
CD138	Phenotypic identification	MCA2459GA	BioRad	Mouse IgG1	B-A38	1:200
CD141	Monocyte subclustering	sc-13164	Santa Cruz Biotechnology	Mouse IgG2a	D-3	1:200
CD163	Phenotypic identification/Monocyte subclustering	ab182422	Abcam	Rabbit	EPR19518	1:227
CD206	Monocyte subclustering	MAB25341	R&D Systems	Mouse IgG2b	#685645	1:250
CD209	Monocyte subclustering	MAB161	R&D Systems	Mouse IgG2b	120507	1:333
Collagen IV	Exploratory Analysis	ab6311	Abcam	Mouse IgG1	COL-94	1:333
FOXP3	Phenotypic identification	ab20034	Abcam	Mouse IgG1	236A/E7	1:1000
Granzyme B	Exploratory Analysis	sc-73620	Santa Cruz Biotechnology	Mouse IgG2a	GRB7	1:50
HLA-DR	Phenotypic identification/Monocyte subclustering	sc-56545	Santa Cruz Biotechnology	Mouse IgG2b	SPM289	1:333

IRF8	Monocyte subclustering	sc-365042	Santa Cruz Biotechnology	Mouse IgG1	E-9	1:400
Ki67	Exploratory Analysis	UM800033	OriGene	Mouse IgG2a	UMAB107	1:5000
LAG3	Exploratory Analysis	#15372	Cell Signaling Technology	Rabbit	D2G4O	1:250
MPO	Phenotypic identification	ab93665	Abcam	Rabbit	SP72	1:1600
OX40	Exploratory Analysis	#61637	Cell Signaling Technology	Rabbit	E9U7O	1:50
PanCK	Phenotypic identification	53-9003-80	ThermoFisher	Mouse IgG1	AE1/AE3	1:1250
PD-1	Exploratory Analysis	#86163	Cell Signaling Technology	Rabbit	D4W2J	1:50
PD-L1	Exploratory Analysis	#13684	Cell Signaling Technology	Rabbit	E1L3N	1:50
PRF1	Phenotypic identification	ab89821	Abcam	Mouse IgG1	5B10	1:20
S100	Phenotypic identification	Z0311	Agilent	Rabbit	Polyclonal	1:4120
TCF7	Exploratory Analysis	#2203	Cell Signaling Technology	Rabbit	C63D9	1:67
TIM3	Exploratory Analysis	AF2365	R&D Systems	Goat	Polyclonal	1:200
AF-488 anti-mouse IgG2a	Secondary antibody	115-545-206	Jackson Immunoresearch	Goat	Polyclonal	5 μg/mL
AF-488 anti-mouse IgG2b	Secondary antibody	115-545-207	Jackson Immunoresearch	Goat	Polyclonal	5 μg/mL
AF-488 anti-mouse IgG3	Secondary antibody	115-545-209	Jackson Immunoresearch	Goat	Polyclonal	5 μg/mL
AF-555 anti-mouse IgG1	Secondary antibody	A-21127	Invitrogen	Goat	Polyclonal	5 μg/mL
AF-555 anti-mouse IgG2a	Secondary antibody	A-21137	Invitrogen	Goat	Polyclonal	5 μg/mL
AF-555 anti-mouse IgG2b	Secondary antibody	A-21147	Invitrogen	Goat	Polyclonal	5 μg/mL
AF-555 anti-goat	Secondary antibody	A-21432	Invitrogen	Donkey	Polyclonal	5 μg/mL
AF-647 anti-rabbit	Secondary Antibody	711-605-152	Jackson Immunoresearch	Donkey	Polyclonal	5 μg/mL

Table S4. Antibodies used for Opal

Marker	Reference	Supplier	Clone
Nkp46	N/A	Innate Pharma	8E5B
CD3	MA1-90582	ThermoFisher	SP7
CD34	GA632	Dako	QBEnd-10
CD163	CD163-L-CE	Leica Biosystem	10D6
