# Supplementary material for: Transcriptional and spatial profiling of the kidney allograft unravels a central role for FcyRIII+ innate immune cells in rejection
Source: Nat Commun. 2023 Jul 19;14:4359. doi: 10.1038/s41467-023-39859-7 (PMC10356785; doi:10.1038/s41467-023-39859-7)
Supplement: Supplementary file 4 — Reporting Summary [file 41467_2023_39859_MOESM4_ESM.pdf]

nature portfolio

Corresponding author(s): Maarten Naesens

Last updated by author(s): Third of January 2023

Reporting Summary

Nature Portfolio wishes to improve the reproducibility of the work that we publish. This form provides structure for consistency and transparency in reporting. For further information on Nature Portfolio policies, see our Editorial Policies and the Editorial Policy Checklist.

Statistics

For all statistical analyses, confirm that the following items are present in the figure legend, table legend, main text, or Methods section.

n/a	Confirmed	
☐	☒	The exact sample size (*n*) for each experimental group/condition, given as a discrete number and unit of measurement
☐	☒	A statement on whether measurements were taken from distinct samples or whether the same sample was measured repeatedly
☐	☒	The statistical test(s) used AND whether they are one- or two-sided<br>*Only common tests should be described solely by name; describe more complex techniques in the Methods section.*
☒	☐	A description of all covariates tested
☐	☒	A description of any assumptions or corrections, such as tests of normality and adjustment for multiple comparisons
☐	☒	A full description of the statistical parameters including central tendency (e.g. means) or other basic estimates (e.g. regression coefficient) AND variation (e.g. standard deviation) or associated estimates of uncertainty (e.g. confidence intervals)
☐	☒	For null hypothesis testing, the test statistic (e.g. *F*, *t*, *r*) with confidence intervals, effect sizes, degrees of freedom and *P* value noted<br>*Give P values as exact values whenever suitable.*
☒	☐	For Bayesian analysis, information on the choice of priors and Markov chain Monte Carlo settings
☒	☐	For hierarchical and complex designs, identification of the appropriate level for tests and full reporting of outcomes
☐	☒	Estimates of effect sizes (e.g. Cohen's *d*, Pearson's *r*), indicating how they were calculated

Our web collection on statistics for biologists contains articles on many of the points above.

Software and code

Policy information about availability of computer code

Data collection	Immunofluorescence images were scanned using the Axio scan.Z1 slidescanner (Zeiss, Germany) at 10X objective with resolution of 0.65 micron/pixel. The hematoxylin and eosin slides were digitized using the Axio scan.Z1 slidescanner in brightfield modus using a 20X objective with resolution of 0.22 micron/pixel.
Data analysis	We used the most recent (as of July 2022) versions of all software programs, including R Studio (version 1.3.1073), SAS (version 9.4, SAS Institute Inc., Cary, NC, United States) and GraphPad Prism (version 9; GraphPad Software, San Diego, CA, United States) for statistical analysis and data presentation. For image analysis, we used R (version 4.2.2) with the following packages: tidyverse version 1.3.1 and EBImage 4.40.0 and QuPath 0.3.2.

For manuscripts utilizing custom algorithms or software that are central to the research but not yet described in published literature, software must be made available to editors and reviewers. We strongly encourage code deposition in a community repository (e.g. GitHub). See the Nature Portfolio guidelines for submitting code & software for further information.

Data

Policy information about availability of data

All manuscripts must include a data availability statement. This statement should provide the following information, where applicable:

- Accession codes, unique identifiers, or web links for publicly available datasets
- A description of any restrictions on data availability
- For clinical datasets or third party data, please ensure that the statement adheres to our policy

All data produced in the present study are available. The Single-cell RNA-sequencing data have been deposited in BioStudies accession code E-MTAB-12051 (https://www.ebi.ac.uk/biostudies/arrayexpress/studies/E-MTAB-12051?accession=E-MTAB-12051). The images generated by MILAN were made available to the reviewers but are not accessible publicly. These image data can be made available upon reasonable request. The kidney transplant biopsy-derived signature matrix encompassing 18 cell types ("KTB18") generated for deconvolution is available in the Source data. This Signature matrix file can be directly used as custom input to run a job within the CIBERSORTx console (https://cibersortx.stanford.edu/runcibersortx.php). Source data are provided with this paper.

Human research participants

Policy information about studies involving human research participants and Sex and Gender in Research.

Reporting on sex and gender	Sex-based analyses (comparison of the expression of sex-related genes within the cells) were performed in order to characterize the origin of the cells whenever a sex mismatch between the organ donor and the allograft recipient would exist (see supplementary figure 3). This study included both male and female participants. Sex annotation was based on self-report. The number of samples for other analyses was too low to allow post-hoc disaggregation of the data by donor and recipient sex.
Population characteristics	The population characteristics were presented in supplementary tables S1 and S2.
Recruitment	Single-cell RNA sequencing was performed on a cohort of 16 biopsies from 14 renal transplant recipients followed in the University Hospitals Leuven, Belgium (Supplementary Table 1). All transplantations were performed with negative complement-dependent cytotoxicity crossmatches on T and B cells. Most recipients had a planned indication biopsy with a high clinical probability for humoral rejection. For two patients, a follow-up biopsy was included in the study.<br>For the multiplex immunofluorescence (MILAN) analysis, an independent set of 18 biopsies was included from renal transplant recipients followed in the University Hospitals Leuven, Belgium (Supplementary Table 2).
Ethics oversight	All patients provided written informed consent. This study was approved by the Ethics Committee of the University Hospitals Leuven (S64904).

Note that full information on the approval of the study protocol must also be provided in the manuscript.

Field-specific reporting

Please select the one below that is the best fit for your research. If you are not sure, read the appropriate sections before making your selection.

☒ Life sciences ☐ Behavioural & social sciences ☐ Ecological, evolutionary & environmental sciences

For a reference copy of the document with all sections, see nature.com/documents/nr-reporting-summary-flat.pdf

Life sciences study design

All studies must disclose on these points even when the disclosure is negative.

Sample size	No sample-size calculation was performed. We considered that 30,000 cells from more than 15 patients were sufficient for single cell RNA sequencing analysis and 500,000 cells from more than 18 patients for single cell immunostaining.
Data exclusions	Data were excluded from the single cells transcriptomic according to the quality threshold based on mitochondrial gene representation as stipulated in supplementary figure 1.
Replication	The main transciptomic finding were replicated in external RNA sequencing datasets (figure 2F) and single cell RNA sequencing (supplementary figure 6). The in vitro experiments were performed at least 3 times independently with different donors. All attempts of replication are shown in the figures.
Randomization	For neighborhood analysis, a quantitative analysis of cell-cell interactions was performed using an adaptation of the algorithm described in Schapiro et al. Briefly, for every cell, all the other cells that are located at a maximum distance d were counted. Then the tissue is randomized preserving the cytometry of the tissue as well as the X and Y coordinates of each cell but permutating the cell identities. This is repeated N times (here N = 1000) which allows to assign an empirical p-value by comparing the number of counts observed in the real tissue versus the number of counts in the randomized cases. Here we performed the described analysis for different values of the distance d (from 10 to 100 micrometers with a step of 10 micrometers) to show the consistency of the reported results. For all other experiments, no randomization was

required.

Blinding | The immunostains were visually evaluated for quality by digital image experts and experienced pathologists (FB, YVH, double blinded). No other quantifications required blinding.

Reporting for specific materials, systems and methods

We require information from authors about some types of materials, experimental systems and methods used in many studies. Here, indicate whether each material, system or method listed is relevant to your study. If you are not sure if a list item applies to your research, read the appropriate section before selecting a response.

Materials & experimental systems

n/a	Involved in the study
☐	☒ Antibodies
☐	☒ Eukaryotic cell lines
☒	☐ Palaeontology and archaeology
☒	☐ Animals and other organisms
☒	☐ Clinical data
☒	☐ Dual use research of concern

Methods

n/a	Involved in the study
☒	☐ ChIP-seq
☐	☒ Flow cytometry
☒	☐ MRI-based neuroimaging

Antibodies

Antibodies used

An overview of the panel with the 38 markers included and the specifications about the primary and secondary antibodies can be found in Supplementary Table 3 for MILAN staining. The 4 markers used in Opal are described in Supplementary Table 4.

For evaluation of intracellular expression of Galectin-9, cells were stained using a fixable viability dye (Fixable Viability Stain 780, BD Biosciences, France) and anti-CD45 BV510 (BD Biosciences) before fixation, permeabilization and staining using an Intracellular staining buffer set (Thermo Fisher Scientific) according to the manufacturer's instructions. Intracellular staining was performed for anti-Galectin-9 FITC (Miltenyi Biotec).

For phenotyping after coculture, cells were incubated with fixable viability dye (Fixable Viability Stain 780) and stained using anti-CD3 BV785 (BD Biosciences), anti-CD8 PerCP-Cy5.5 (BioLegend), anti-CD14 BV510 (BD Biosciences) and anti-CD107a FITC (Thermo Fisher Scientific) antibodies. The cells were subsequently fixed and permeabilized (Cytofix/Cytoperm fixation/permeabilization kit; BD Biosciences), stained with anti-Granzyme-B AlexaFluor700 (BD biosciences) and anti-IFNg PE (BD biosciences) antibodies and analyzed by flow cytometry.

Validation

All antibodies used herein are commercially available and have been validated by their suppliers.

MILAN Antibodies:

AQP1 https://www.sigmaaldrich.com/FR/fr/product/mm/ab2219
CD1c https://www.origene.com/catalog/antibodies/primary-antibodies/um500042/cd1c-mouse-monoclonal-antibody-clone-id-umab46
CD3 https://www.thermofisher.com/antibody/product/CD3e-Antibody-clone-SP7-Monoclonal/MA1-90582
CD4 https://www.abcam.com/products/primary-antibodies/cd4-antibody-epr6855-ab133616.html
CD8 https://www.scbt.com/p/cd8-alpha-antibody-c8-144b
CD11b https://www.abcam.com/products/primary-antibodies/cd11b-antibody-epr1344-ab133357.html
CD11c https://www.scbt.com/p/integrin-alphax-antibody-b-6
CD14 https://www.cellsignal.com/products/primary-antibodies/cd14-d7a2t-rabbit-mab-ihc-formulated/75181
FcgIII https://shop.leicabiosystems.com/fr-fr/ihc-ish/ihc-primary-antibodies/pid-cd16
CD20 https://www.agilent.com/store/fr_FR/Prod-M075501-2/M075501-2
CD31 https://www.lsbio.com/antibodies/ihc-plus-pecam-1-antibody-cd31-antibody-clone-oti2c6-ihc-wb-western-ls-b16850/856292
CD56 https://www.scbt.com/p/ncam-antibody-123c3
CD57 https://www.rndsystems.com/products/human-beta-13-glucuronyltransferase-1-b3gat1-antibody-1002707_mab8560
CD68 https://www.thermofisher.com/antibody/product/CD68-Antibody-clone-PG-M1-Monoclonal/MA5-12407
CD69 https://www.sigmaaldrich.com/FR/fr/product/sigma/hpa050525
CD79a https://www.scbt.com/p/cd79a-antibody-jcb117
CD123 https://shop.leicabiosystems.com/fr-fr/ihc-ish/ihc-primary-antibodies/pid-cd123
CD138 https://www.bio-rad-antibodies.com/monoclonal/human-cd138-antibody-b-a38-mca2459.html?f=purified&JSESSIONID_STERLING=1A7EACE163A2E349333EBF2B6A85B3B4.ecommerce1&evCntryLang=FR-fr&cntry=FR&thirdPartyCookieEnabled=true
CD141 https://www.scbt.com/p/tm-antibody-d-3
CD163 https://www.abcam.com/products/primary-antibodies/cd163-antibody-epr19518-bsa-and-azide-free-ab213612.html
CD206 https://www.rndsystems.com/products/human-mmr-cd206-antibody-685645_mab25341#product-citations
CD209 https://www.rndsystems.com/products/human-dc-sign-cd209-antibody-120507_mab161

Collagen IV https://www.abcam.com/products/primary-antibodies/collagen-iv-antibody-col-94-ab6311.html
FOXP3 https://www.abcam.com/products/primary-antibodies/foxp3-antibody-236ae7-ab20034.html
GranzympeB https://www.scbt.com/p/granzyme-b-antibody-grb7
HLA-DR https://www.scbt.com/p/hla-dr-antibody-spm289
IRF8 https://www.scbt.com/p/icsbp-antibody-e-9
Ki67 https://www.origene.com/catalog/antibodies/primary-antibodies/um800033/ki67-mki67-mouse-monoclonal-antibody-clone-id-umab107
LAG3 https://www.cellsignal.com/products/primary-antibodies/lag3-d2g4o-xp-rabbit-mab/15372
MPO https://www.abcam.com/products/primary-antibodies/myeloperoxidase-antibody-sp72-ab93665.html
OX40 https://www.cellsignal.com/products/primary-antibodies/ox40-e9u7o-xp-rabbit-mab/61637
PanCK https://www.thermofisher.com/antibody/product/Pan-Cytokeratin-Antibody-clone-AE1-AE3-Monoclonal/53-9003-82
PD1 https://www.cellsignal.com/products/primary-antibodies/pd-1-d4w2j-xp-rabbit-mab/86163
PDL1 https://www.cellsignal.com/products/primary-antibodies/pd-l1-e1l3n-xp-rabbit-mab/13684?Ntt=13684&_=1666110429376&tahead=true&utm_region=hq&utm_seg=ind&utm_stage=ous&utm_tactic=ppc&utm_prog=rvg&utm_conv=tdr&utm_source=google&utm_medium=cpc&utm_campaign=bpa&utm_content=22-bpa-29633&gclid=Cj0KCQjwjryjBhD0ARIsAMLvnF8KErgbSZ6zjq3BO-gm82clkhcw7V9L9vSqYeW-bkNYPBPSHZ4UQxYaAiMiEALw_wcB&gclsrc=aw.ds
PRF1 https://www.abcam.com/products/primary-antibodies/perforin-antibody-5b10-ab89821.html
S100 https://www.agilent.com/en/product/immunohistochemistry/antibodies-controls/primary-antibodies/s100-(dako-omnis)-76198
TCF7 https://www.cellsignal.com/products/primary-antibodies/tcf1-tcf7-c63d9-rabbit-mab/2203
TIM3 https://www.rndsystems.com/products/human-tim-3-antibody_af2365
AF-488 anti-mouse IgG2a https://www.jacksonimmuno.com/catalog/products/115-545-206
AF-488 anti-mouse IgG2b https://www.jacksonimmuno.com/catalog/products/115-545-207
AF-488 anti-mouse IgG3 https://www.jacksonimmuno.com/catalog/products/115-545-209
AF-555 anti-mouse IgG1 https://www.thermofisher.com/antibody/product/Goat-anti-Mouse-IgG1-Cross-Adsorbed-Secondary-Antibody-Polyclonal/A-21127
AF-555 anti-mouse IgG2a https://www.thermofisher.com/antibody/product/Goat-anti-Mouse-IgG2a-Cross-Adsorbed-Secondary-Antibody-Polyclonal/A-21137
AF-555 anti-mouse IgG2b https://www.thermofisher.com/antibody/product/Goat-anti-Mouse-IgG2b-Cross-Adsorbed-Secondary-Antibody-Polyclonal/A-21147
AF-555 anti-goat https://www.thermofisher.com/antibody/product/Donkey-anti-Goat-IgG-H-L-Cross-Adsorbed-Secondary-Antibody-Polyclonal/A-21432
AF-647 anti-rabbit https://www.jacksonimmuno.com/catalog/products/711-605-152

Opal antibodies

Nkp46 https://www.innate-pharma.com/sites/default/files/iph2201_mona_cetux_posterpreclin_bat.pdf
CD3 https://www.thermofisher.com/antibody/product/CD3e-Antibody-clone-SP7-Monoclonal/MA1-90582
CD34 https://www.agilent.com/en/product/immunohistochemistry/antibodies-controls/primary-antibodies/cd34-class-ii-(dako-omnis)-76244
CD163 https://shop.leicabiosystems.com/fr-fr/ihc-ish/ihc-primary-antibodies/pid-cd163

Flow cytometry antibodies
CD45-BV510 https://www.bdbiosciences.com/en-nl/products/reagents/flow-cytometry-reagents/research-reagents/single-color-antibodies-ruo/bv510-mouse-anti-human-cd45.563204
Galectin9-FITC https://www.miltenyibiotec.com/US-en/products/galectin-9-antibody-anti-human-reafinity-rea435.html#conjugate=fitc:size=100-tests-in-200-ul
CD3-BV786 https://www.bdbiosciences.com/en-us/products/reagents/flow-cytometry-reagents/research-reagents/single-color-antibodies-ruo/bv786-mouse-anti-human-cd3.565491
CD8-PerCP-Cy5.5 https://www.biolegend.com/de-at/products/percp-cyanine5-5-anti-human-cd8-antibody-6389?GroupID=BLG10167
CD14-BV510 https://www.bdbiosciences.com/en-au/products/reagents/flow-cytometry-reagents/research-reagents/single-color-antibodies-ruo/bv510-mouse-anti-human-cd14.563079
CD107a-FITC https://www.thermofisher.com/antibody/product/CD107a-LAMP-1-Antibody-clone-eBioH4A3-Monoclonal/11-1079-42
GranzymeB-AF700 https://www.bdbiosciences.com/en-ca/products/reagents/flow-cytometry-reagents/research-reagents/single-color-antibodies-ruo/alexa-fluor-700-mouse-anti-human-granzyme-b.560213
IFNg-PE https://www.bdbiosciences.com/en-nz/products/reagents/flow-cytometry-reagents/research-reagents/single-color-antibodies-ruo/pe-mouse-anti-human-ifn.554552

Eukaryotic cell lines

Policy information about cell lines and Sex and Gender in Research

Cell line source(s)	ATCC
Authentication	HK2 cell line was supplied from ATCC and is NOT on the latest version of ICLAL list of misidentified cell lines
Mycoplasma contamination	Cell lines used were tested for mycoplasma contamination and confirmed negataive of infection via PCR

Commonly misidentified lines (See ICLAC register)	Not relevant

Flow Cytometry

Plots

Confirm that:

- [x] The axis labels state the marker and fluorochrome used (e.g. CD4-FITC).
- [x] The axis scales are clearly visible. Include numbers along axes only for bottom left plot of group (a 'group' is an analysis of identical markers).
- [x] All plots are contour plots with outliers or pseudocolor plots.
- [x] A numerical value for number of cells or percentage (with statistics) is provided.

Methodology

Sample preparation	For evaluation of intracellular expression of Galectin-9, cells were stained using a fixable viability dye (Viobility Dye, Miltenyi Biotec) and anti-CD45 BV510 (BD Biosciences, France) before fixation, permeabilization and staining using an Intracellular staining buffer set (Thermo Fisher Scientific) according to the manufacturer’s instructions. Intracellular staining was performed for anti-Galectin-9 FITC (Miltenyi Biotec).
Instrument	Cells were analyzed using a Attune analyzer (Thermo Fisher Scientific).
Software	Cells were analyzed with FlowJo software (BD Biosciences)
Cell population abundance	All sorted cell populations exhibited high purity (>90%), as revealed by flow cytometry
Gating strategy	Cells were gated using FSC/SSC parameters then singlets were gated and living cells (FVD negative). To distinguish monocytes from endothelial cells, CD45 staining was used and percentage of galectin postive cells were determined in CD45+ cells.

- [x] Tick this box to confirm that a figure exemplifying the gating strategy is provided in the Supplementary Information.
